# Feasibility and safety of targeting mitochondria for cancer therapy – preclinical characterization of gamitrinib, a first-in-class, mitochondriaL-targeted small molecule Hsp90 inhibitor

**DOI:** 10.1080/15384047.2022.2029132

**Published:** 2022-02-06

**Authors:** Umar Hayat, Gary T. Elliott, Anthony J. Olszanski, Dario C. Altieri

**Affiliations:** a Pharmaceutical Advisors, LLC, Princeton, USA; b Galenic Strategies Inc, Windsor; c Phase 1 Developmental Therapeutics Program, Department of Hematology/Oncology Fox Chase Cancer Center, Philadelphia; d Immunology, Microenvironment and Metastasis Program, The Wistar Institute, Philadelphia, USA

**Keywords:** Mitochondria, cancer therapy, Hsp90, Gamitrinib

## Abstract

Mitochondria are key tumor drivers, but their suitability as a therapeutic target is unknown. Here, we report on the preclinical characterization of Gamitrinib (*GA mit*ochondrial ma*tri*x *in*h*ib*itor), a first-in-class anticancer agent that couples the Heat Shock Protein-90 (Hsp90) inhibitor 17-allylamino-geldanamycin (17-AAG) to the mitochondrial-targeting moiety, triphenylphosphonium. Formulated as a stable (≥24 weeks at −20°C) injectable suspension produced by microfluidization (<200 nm particle size), Gamitrinib (>99.5% purity) is heavily bound to plasma proteins (>99%), has intrinsic clearance from liver microsomes of 3.30 mL/min/g and minimally penetrates a Caco-2 intestinal monolayer. Compared to 17-AAG, Gamitrinib has slower clearance (85.6 ± 5.8 mL/min/kg), longer t_1/2_ (12.2 ± 1.55 h), mean AUC_0-t_ of 783.1 ± 71.3 h∙ng/mL, and unique metabolism without generation of 17-AG. Concentrations of Gamitrinib that trigger tumor cell killing (IC_50_ ~1-4 µM) do not affect cytochrome P450 isoforms CYP1A2, CYP2A6, CYP2B6, CYP2C8 or ion channel conductance (Nav1.5, Kv4.3/KChIP2, Cav1.2, Kv1.5, KCNQ1/mink, HCN4, Kir2). Twice weekly IV administration of Gamitrinib to Sprague-Dawley rats or beagle dogs for up to 36 d is feasible. At dose levels of up to 5 (rats)- and 12 (dogs)-fold higher than therapeutically effective doses in mice (10 mg/kg), Gamitrinib treatment is unremarkable in dogs with no alterations in clinical-chemistry parameters, heart function, or tissue histology, and causes occasional inflammation at the infusion site and mild elevation of serum urea nitrogen in rats (≥10 mg/kg/dose). Therefore, targeting mitochondria for cancer therapy is feasible and well tolerated. A publicly funded, first-in-human phase I clinical trial of Gamitrinib in patients with advanced cancer is ongoing (ClinicalTrials.gov NCT04827810)

## Introduction

The rewiring of metabolic pathways, including in mitochondria,^1^ is a ubiquitous cancer trait important for disease progression.^2^ Accordingly, exploitation of mitochondrial bioenergetics,^3^ buffering of reactive oxygen species (ROS),^4^ and inhibition of cell death pathways^5^ have been implicated in tumor growth, acquisition of metastatic competence, and resistance to conventional and molecular therapy.^6^ On this basis, mitochondria may provide a unique therapeutic target for cancer,^7,8^ suitable to disable key pathways for tumor maintenance, regardless of genetic makeup or driver mutation(s). As a result, multiple mitochondrial-targeted anticancer agents, *or mitocans*,^9^ have been developed, and mitochondrial protease ClpP agonist, ONC201,^10^ Complex I inhibitor metformin/ME-344,^11^ antagonist of mitochondrial translation, tigecycline,^12^ and PDH/KDH blocker CPI-613^13^ have entered clinical trials in humans.

In addition, there is evidence that mitochondria may be uniquely ‘wired’ in cancer compared to normal tissues, potentially enabling a broader therapeutic window.^14^ One example is the ubiquitous dependence or ‘addiction’ of tumor mitochondria to a heightened protein folding environment,^15^ essential to buffer the proteotoxic stress invariably associated with tumor growth, in vivo.^16^ Mechanistically, this is accomplished by the selective accumulation of molecular chaperones, including Heat Shock Protein-90 (Hsp90) and its homolog TNF Receptor-Associated Protein 1 (TRAP1),^17^ as well as AAA+ proteases^18^ in tumor mitochondria, compared to normal tissues. In turn, Hsp90 chaperoning stabilizes the multifunctional mitochondrial proteome in cancer,^19,20^ including key metabolic regulators,^21^ lowers ROS,^22^ and prevents cell death.^17,23,24^

Although targeting chaperone-directed proteostasis in mitochondria shows promising antitumor activity,^25,26^ pharmacologically, this pathway escapes inhibition by small-molecule Hsp90 antagonists with geldanamycin (GA) or non-GA backbones^27^ as these agents fail to accumulate in mitochondria.^28^ To overcome this conundrum, we generated Gamitrinib (*GA mit*ochondrial ma*tri*x *in*h*ib*itor), a first-in-class, mitochondrial-targeted inhibitor of organelle protein folding^25^ that links the GA Hsp90 inhibitor 17-allylamino-geldanamyicn (17-AAG, Tanespimycin)^27^ to an efficient mitochondrial-import carrier, triphenylphosphonium (TPP).^29^ Due to its unique chemical structure, Gamitrinib selectively accumulates in mitochondria^28^ with a 106-fold enrichment compared to cytosol by mass spectrometry of isolated subcellular fractions.^30^ Once in mitochondria, Gamitrinib triggers acute proteotoxic stress,^20,23^ shuts off multipleorganelle functions, including bioenergetics,^19^ and delivers potent anticancer activity with IC_50_ of 0.16–29 µM in an NCI 60 cell-line screen, including cell lines representative of common malignancies, such as colon adenocarcinoma (IC_50_, 0.35–29 µM), breast adenocarcinoma (IC_50_, 0.16–3.3 µM), and melanoma (IC_50_, 0.36–2.7 µM).^31,32^ Strong anticancer activity was also seen in a combination of regimens of Gamitrinib plus molecular therapy^33–35^ in models of epithelial and hematopoietic malignancies.

Here, we report the preclinical characterization of Gamitrinib as the first, subcellularly directed antagonist of mitochondrial proteostasis. Based on these findings, a publicly funded, first-in-human phase I clinical trial of Gamitrinib in patients with advanced cancer is currently ongoing (ClinicalTrials.gov, NCT04827810).

## Materials and methods

### Cell lines

Caco-2 and HEK293 cells were obtained from the American Type Culture Collection (ATCC, Manassas, VA) and grown in culture according to the supplier’s specifications. In some experiments, HEK293 cells were stably transfected with hERG cDNA, and polyclonal cultures were maintained in the presence of 250 µg/ml geneticin (G418). Cells were maintained in a medium containing 10% fetal calf serum at 37°C in 5% CO_2_ and plated on 35 mm dishes at least 24 h prior to the experiment.

### Chemical synthesis and analysis of Gamitrinib

The complete chemical synthesis, HPLC profile, and mass spectrometry of Gamitrinib (*GA mit*ochondrial ma*tri*x *in*h*ib*itor) have been described previously.^28^ The structure of Gamitrinib is combinatorial and contains the Hsp90 inhibitor 17-AAG linked to triphenylphosphonium as a mitochondrial-targeting carrier. The bulk Gamitrinib powder is stored at −20°C in the dark.

### Formulation development of Gamitrinib

A sequential three-step process was utilized to prepare Good Laboratory Practice (GLP) working solutions of Gamitrinib (5 mg/mL) for preclinical studies. The formulation workflow is as follows: Step 1 – Solubilization of Gamitrinib powder in DMSO (2.5%); Step 2 – Dilution in 1.25% (w/v) Polysorbate 80, 0.31% (w/v) Lecithin (Lipoid S100) and 12.5% (w/v) Sucrose (10%) in sterile water for injection; Step 3 – Dilution in 5% dextrose (87.5%). Therefore, the final Gamitrinib formulation is ~5 mg/mL Gamitrinib, 2.5% DMSO, 0.125% Polysorbate 80, 0.031% Lecithin, 1.25% Sucrose, and 4.375% Dextrose. For Good Manufacturing Practice (GMP) studies, a Gamitrinib Injectable Suspension (GIS) was prepared by microfluidization. Gamitrinib stock solutions prepared as above were passed through a microfluidizer (Dyhydromatics, Acton, MA) with the rate of flow set at low, medium, and high. At the end of GIS processing, microfluidization was continued at reduced pressure (~2000 psi) for 1–2 min. The parameters for GIS microfluidization as are follows: ratio of organic to aqueous phase (DMSO: aqueous vehicle 1:40 v/v); filter membrane materials (PTFE for DMSO, cellulose acetate for aqueous vehicle); microfluidizer pressure during mixing organic and aqueous phase (28,000 psi); post-mixing pressure in microfluidizer (2000 psi); temperature inside interaction chamber (0 to −10°C before initiating microfluidization). The final GIS after microfluidization is 4.86 mg/mL, with average particle size of 154 nm, D(0.9) size of 229 nm, pH 6.0.

### Gamitrinib pharmacokinetics (PK) in rat plasma

Sprague-Dawley rats (n = 3) were administered with a single intravenous (IV) dose of 1 mL Gamitrinib (5 mg/kg) formulated as described above without added microfluidization. Blood samples were collected via lateral tail vein using K_2_EDTA as an anticoagulant at 0.083, 0.25, 0.5, 1, 2, 4, and 24 h post-dose, chilled on ice, and centrifuged at 5000 rpm for 10 min. Aliquots (0.2 mL) of each plasma sample were stored frozen at −20°C. For analysis, 50 µL aliquots of the study samples were mixed with 50 µL of ACN/H_2_O (50:50), extracted by protein precipitation using ACN containing the internal standard Gamitrinib-d_15_ (100 ng/mL), and the aliquots of the supernatant were mixed with deionized water for Gamitrinib determination. Batch samples under analysis included a calibration curve, a matrix blank (blank rat plasma), a reagent blank, a control zero (blank rat plasma spiked with internal standard), and duplicate QC samples at three concentration levels (low, medium, and high) in addition to the study samples. Within each batch, the study samples were bracketed by calibration standards or QC samples. The lowest calibration standard served to evaluate system suitability at the beginning of each batch. In all batches, the system suitability samples displayed adequate separation and acceptable peak shapes, retention times, and signal-to-noise ratios. Overall, the concentration of Gamitrinib was measured in 21 rat plasma samples in 1 analytical batch using LC-MS/MS data acquisition on a Shimadzu Nexera LC system coupled with an AB Sciex Triple Quad 5500 mass spectrometer. Chromatograms were integrated using Analyst 1.6.2 software. A weighted (1/*x*^2^, *x* = concentration) linear regression was used to generate the calibration curve for Gamitrinib. The concentration of Gamitrinib was calculated using the peak area ratio of analyte to internal standard based on the standard curve. The mean, standard deviation, precision, accuracy, and assay variability were calculated using Microsoft Excel.

### Toxicity in rats

Ninety-six male and female Sprague-Dawley rats (6–7 weeks old) were received from Charles River Laboratories (Raleigh, NC) catheterized via a femoral vein prior to arrival. Animals were acclimated in individual stainless-steel cages with access to water and Certified Rodent Diet #2014 C (Envigo RMS) *ad libitum* for 6 d prior to study initiation. Environmental controls were set to maintain a temperature range of 20 to 26°C, a relative humidity range of 30 to 70%, eight or greater air changes/h, and a 12-h light/dark cycle. Animals were infused with sterile isotonic (0.9%) saline for a minimum of 4 d (0.20 mL/h for males or 0.15 mL/h for females) during the predose phase. At initiation of dosing, animals were 8 to 9 weeks old, and body weights ranged from 283 to 341 g for males and 188 to 238 g for females. Gamitrinib formulated as indicated above was administered by IV infusion on d 4, 8, 11, 15, 18, 22, 25, and 29 of the dosing phase at 1, 10, 25 mg/kg/dose and dose volume of 5 mL/kg. The vehicle control administered with the same schedule contained 2.5% DMSO, 0.125% polysorbate 80, 0.031% lecithin, 1.25% sucrose and 4.375% dextrose. Animals were checked twice daily throughout the duration of the study for mortality, abnormalities, and signs of pain or distress. Detailed observations were conducted for each animal up to two times during the predose phase and for each toxicity animal prior to dosing on d 1, 8, 15, 22, and 29 of the dosing phase and on d 1, 7, and 14 of the recovery phase. Blood samples were collected on d. 1, 4 and 29 of the dosing phase 5 min, 1 h and 24 h post-dose, maintained on chilled cryoracks and centrifuged within 1 h of collection. Tissue samples harvested from each animal were embedded in paraffin, sectioned, and slides were prepared and stained with hematoxylin and eosin.

### Toxicity in beagle dogs

Eighteen male and female (5–6 mo old) purebred beagle dogs (Cumberland, VA) were acclimated for 47 d (20°C to 26°C, relative humidity 30–70%, 10 or greater air changes/h, and a 12-h light/dark cycle) prior to study initiation. Animals were given water *ad libitum* and Certified Canine Diet #5007 (PMI Nutrition International Certified LabDiet®) for 4 to 5 h each day. At study initiation, animal body weights ranged from 8.6 to 11.5 kg for males and 6.0 to 9.4 kg for females. At least 1 week prior to initiation of dosing, animals were fasted overnight, anesthetized, and a catheter attached to a subcutaneous vascular access port was surgically implanted into a jugular vein. Animals were acclimated to infusion jackets prior to catheter implantation surgery. Animals were infused with sterile saline at a dose rate of 5 mL/h when the vascular access ports were accessed and when animals were connected to the infusion system (except when dosed with the test or vehicle control article). The patency of each catheter was checked as needed. Prednisolone tablets were administered orally at 2 mg/kg the night prior to dosing. Diphenhydramine (5 mg/kg; IM injection 0.1 mL/kg, 50 mg/mL) was administered prior to dosing and post the start of infusion (d 1 only). Male and female dogs received dose levels of Gamitrinib of 1.25, 3.33 and 6.25 mg/kg/dose formulated as indicated above during a 1 h infusion on d 1, 8, 11, 15, 18, 22, 25, 29, 32, and 36 at a volume of 2 mL/kg. The vehicle control was as above. During dosing on d 1, multiple animals, including controls, were noted with clinical observations of swollen head/body, hypoactivity, twitching, red skin, and/or vocalization due to vehicle components. Dosing was stopped, and 23 of 24 animals were administered additional diphenhydramine, 11 were administered Flunixin meglumine and buprenorphine, and 6 were administered acepromazine. Symptoms subsided in all animals after completion of these interventions. As a result of these observations, the polysorbate levels, which were increased from 0.025% (w/v) to 0.125% (w/v) to minimize precipitation, were returned to the original concentrations; 2 mg/kg prednisone was administered orally the night prior to dosing; and the diphenhydramine pretreatment dose was updated to 5 mg/kg 15 to 30 min prior to dosing. Detailed observations were conducted for each animal up to seven times during the predose phase, prior to dosing on d 1, 8, 15, 22, 29, and 36 of the dosing phase, and on d 1, 7, and 14 of the recovery phase. Blood samples (1 mL) were collected via the cephalic vein 5, 15, and 30 min and 1, 4, 8, and 24 h post the end of infusion on d 8 and 36 of the dosing phase. Formalin-fixed and paraffin-embedded tissue samples collected at the end of the study (d 39) were processed for histologic examination.

### Statistical analysis

Levene’s test was used to test for equality of variances between groups. Where Levene’s test was not significant (P > .05), ANOVA was conducted; where Levene’s test was significant (P ≤ .05), a rank transformation was applied before the ANOVA was conducted. Where the group effect from the ANOVA was significant (P ≤ .05), comparisons between each treated group and the control were made using Dunnett’s t-test. If the ANOVA was not significant (P > .05), no further analyses were conducted.

## Results

### Chemical synthesis and formulation development of Gamitrinib

The chemical synthesis of Gamitrinib containing the Hsp90 inhibitor 17-AAG linked to the mitochondrial-targeting carrier, triphenylphosphonium via a hexylamine linker has been described.^28^ Clinical-grade (GMP compliant) Gamitrinib synthesized as described in Supplementary Figure S1A^28^ has the chemical formula C_52_H_65_F_6_N_3_O_8_P_2_ (>99.5% purity by UPLC), is purple solid (TM.795) and crystalline by X-ray powder diffraction, with a molecular weight of 1036.03. A 500 MHz^1^H NMR spectrum (DMSO-*d6*), 125 MHz^13^C NMR spectrum (DMSO-*d6*) and 282 MHz^19^F NMR spectrum (DMSO-*d6*) are all consistent with structure. The water content is 0.7% (Karl Fischer analysis), and the residual solvent concentrations (methanol, DCM, MTBE, and DIPEA) are all below limit of quantification (BLOQ, <3000, <600, <5000, and <3000 ppm, respectively). Gamitrinib is formulated using a three-step dilution process described in the Materials and Methods section. A sterile, Gamitrinib Injectable Suspension (GIS) is prepared for clinical use using microfluidization with the schematic flowchart shown in Supplementary Figure S1B. The resulting GIS has average particle size of 154 nm and D(0.9) size of 229 nm, pH 6.0. When stored frozen at −20°C, the GIS shows no significant changes in stability or particle size distribution upon analysis at 1, 2, 4, 8, 12, and 24 weeks after manufacturing (Table 1).

**Table 1.** Stability and particle size distribution of Gamitrinib Injectable Suspension (GIS)

	Time of storage (2 ml GIS in 4 ml sterile glass vials at −20°C						
	0	1 wk	2 wk	4 wk	8 wk	12 wk	24 wk
Appearance	Purple susp	Purple susp	Purple susp	Purple susp	Purple susp	Purple susp	Purple susp
Particle size (nm)	200	198	204	203	205	203	212
F/T particle size (nm)		197	209	207	216	208	225
Assay (mg/ml)	4.41	4.80	4.57	4.43	4.47	4.47	4.61
Recovery (% over T0)	100	109	103	100	101	101	104

Susp, suspension; F/T, freeze-thaw; wk, week.

### In vitro toxicity

Concentrations of Gamitrinib that trigger tumor cell killing in culture (IC_50_ ~1–4 µM)^28,30^ did not inhibit cytochrome P450 isoforms CYP1A2 (IC_50_, 32.9 µM), CYP2A6 (IC_50_, 24 µM), CYP2B6 (IC_50_, 16 µM), and CYP2C8 (IC_50_, 8 µM) (Supplementary Figure S2). Conversely, Gamitrinib inhibited CYP2C9 (IC_50_, 1.1 µM) and CYP3A4 (IC_50_, 0.12–0.2 µM) (Supplementary Figure S2).

**Figure 1.**
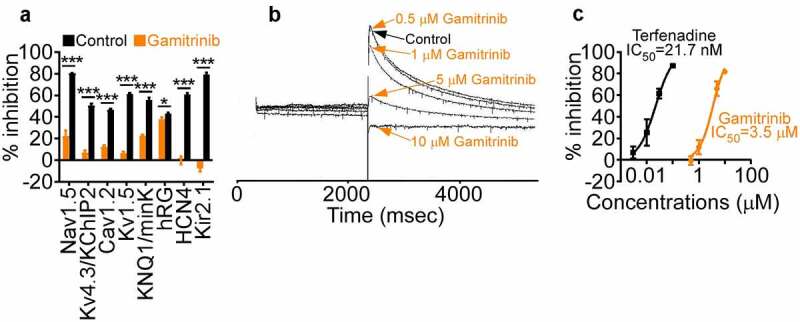
Ion channel activity. (a) Gamitrinib (10 µM) or relevant control was incubated with the individual channel-containing samples and the % inhibition of conductance compared to control was quantified. Mean ± SD. *, p = .02; ***, p < .0001. (b) Recording of hERG currents in the presence of control or the indicated increasing concentrations of Gamitrinib. Representative experiment. (c) HEK293 cells were transfected with hERG and analyzed for inhibition of hERG currents in the presence of increasing concentrations of Gamitrinib or control terfenadine. The IC_50_ values for each compound tested are indicated.

When analyzed for ion channel conductance, high concentrations of Gamitrinib (10 µM) inhibited Nav1.5 currents by 22.3 ± 5.3% (control, 80.3 ± 0.5%; n = 3, pulse 26), Kv4.3/KChIP2 by 6.8 ± 2.2% (control 50.5 ± 2; n = 13), Cav1.2 by 12.2 ± 1.5% (control, 46.8 ± 0.7%; n = 34, pulse 2), Kv1.5 by 6.6 ± 1.3% (control 61 ± 1%; n = 15), KCNQ1/mink by 22.5 ± 1.1% (control 55.7 ± 2.4%; n = 16), hERG by 37.9 ± 1.7% (control 42.9 ± 1.2%; n = 16), HCN4 by −0.2 ± 3.7% (control 60.8 ± 1.2%; n = 16), and Kir2.1 by −7.3 ± 3.4% (control 79.2 ± 1.9%; n = 15, pulse 10) (Figure 1(a)). The potential effect of Gamitrinib on hERG currents was further studied in patch-clamp experiments in transfected HEK293 cells (Figure 1(b)). Here, concentrations of Gamitrinib of 0.5, 1, 5, and 10 µM inhibited hERG currents by −0.68 ± 3.95%, 11.71 ± 6.51%, 65.95 ± 6.78%, and 81.83 ± 1.88%, respectively (mean ± SD) (Figure 1(b)), resulting in a Gamitrinib IC_50_ of hERG inhibition of 3.5 µM (terfenadine IC_50_ 21.7 nM) (Figure 1(c)).

To further characterize the potential cardiac toxicity of Gamitrinib, electrocardiography studies were carried out on beagle dogs administered IV Gamitrinib at dose levels of 1.25, 3.3, and 6.25 mg/kg/dose twice weekly for 36 d plus a 14-d recovery period (Supplementary Figure S3). In this analysis, one out of five male dogs administered Gamitrinib at 6.25 mg/kg/dose exhibited a small (7%) prolongation of QTc interval (17 msec) on d 32 of the dosing phase, which reversed during the recovery phase. No Gamitrinib-related prolongation of QTc interval was observed in female dogs administered 6.25 mg/kg/dose or in both sexes administered 1.25 or 3.33 mg/kg/dose (Supplementary Figure S3). No Gamitrinib-related ECG changes in PR interval, QRS duration, QT interval, or heart rate were observed on d 32 of the dosing phase in animals administered 1.25, 3.33, or 6.25 mg/kg/dose or on d 11 of the recovery phase in animals administered 6.25 mg/kg/dose. No other rhythm abnormalities or qualitative ECG changes were observed (Supplementary Figure S3).

### PK studies

**Figure 2.**
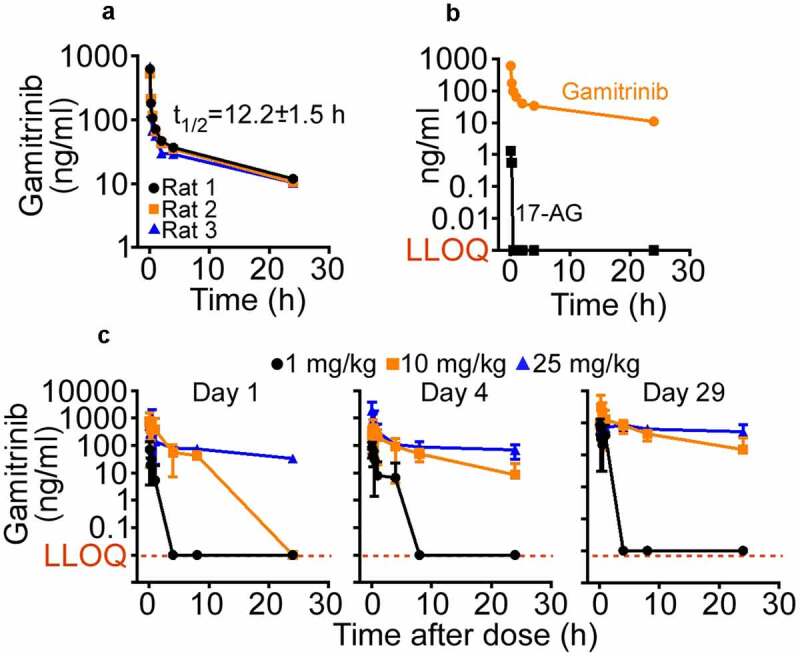
Gamitrinib PK in rats. (a) Gamitrinib (5 mg/kg) was injected IV in Sprague-Dawley rats and blood samples collected at the indicated time intervals were analyzed for Gamitrinib concentrations (C_max_). Data from three individual animals and t_1/2_ values (mean ± SD) are shown. (b) The conditions are as in (A) and plasma samples from rats administered IV Gamitrinib were analyzed for Gamitrinib or 17-AG concentrations. LLOQ, lower limit of quantification. (c) Male and female Sprague-Dawley rats administered Gamitrinib IV at three dose levels (1, 10 and 25 mg/kg/dose) twice weekly were analyzed for Gamitrinib concentrations (C_max_) on d 1, 4 and 29 of the dosing phase (mean ± SD). LLOQ, lower limit of quantification.

After the IV administration (5 mg/kg) to Sprague-Dawley rats (n = 3), the mean Gamitrinib C_max_ was 1175.807 ng/mL (Figure 2(a)), with a mean volume of distribution at steady state (V_ss_) of 65.471 L/kg, medium to slow clearance at 85.656 ± 5.856 ml/min/kg, and mean terminal-phase half-life (t_1/2_) of 12.25 ± 1.55 h (Table 2). Mean AUC_0-t_ and AUC_INF_ values were 783.199 and 976.002 h∙ng/mL, respectively (Table 2). Gamitrinib metabolism in rats did not generate detectable levels of 17-(amino)-17-demethoxygeldanamycin (17-AG) (Figure 2(b), Supplementary Table S2), a key metabolite of 17-AAG processing, in vivo.^36^ The IV administration of Gamitrinib to Sprague-Dawley rats at dose levels of 1, 10, or 25 mg/kg/dose twice weekly for 29 d resulted in increased C_max_ values from 1 to 25 mg/kg/dose followed by a bi-exponential decline (Figure 2(c)). CL_SS_ values ranged from 84.83 to 131.33 mL/min/kg and V_SS_ values from 12.2 to 90.0 L/kg for d 4 and d 29. Gamitrinib C_max_ and AUC_0–24_ values were similar on d 4 and d 29, indicating no drug accumulation after multiple doses. Accumulation ratio values ranged from 0.045 to 1.04 for C_max_ and from 0.344 to 1.33 for AUC_0–24_.

**Table 2.** Gamitrinib (5 mg/kg IV) PK in Sprague-Dawley rats

PK Parameters	Unit	Rat 1	Rat 2	Rat 3	Mean	SD	CV (%)
*t*_1/2_	h	11.623	11.107	14.022	12.250	1.555	12.7
C_max_	ng/mL	1153.712	829.668	1544.041	1175.807	357.699	30.4
CL	mL/min/kg	79.233	87.035	90.700	85.656	5.856	6.8
MRT	h	5.539	5.411	5.524	5.492	0.0702	1.3
Vz	L/Kg	79.715	83.678	110.085	91.159	16.509	18.1
Vss	L/Kg	58.230	60.052	78.131	65.471	11.001	16.8
AUC_last_	h∙ng/mL	851.441	789.045	709.112	783.199	71.344	9.1
AUC_INF_	h∙ng/mL	1051.752	957.472	918.784	976.002	68.394	7.0

AUC_INF_; area under concentration vs. time curve from time 0 to infinity; AUC_last_, area under concentration vs. time curve from time 0 to last quantifiable concentration; C_max_, maximum-observed concentration; CL, clearance; MRT, mean residence time; t_1/2_, terminal half-life; Vz, apparent volume of distribution; Vss, volume of distribution at steady-state.

### Plasma protein binding, stability, microsome clearance, and intestinal penetration

Gamitrinib was heavily bound to plasma proteins (99.3 ± 0.07%) with an average free fraction of 0.7 ± 0.07%, comparable to control warfarin (bound, 98.3 ± 0.22%, free fraction, 1.68 ± 0.22%). The stability of Gamitrinib in human plasma was 91.4% with an average recovery of 82.8 ± 3% (warfarin, 88.3 ± 2.9%). At a concentration of 0.5 µM, the elimination rate constant (k) of Gamitrinib in phase I, cytochrome P450-mediated human liver microsome metabolism was 0.041 (control Midazolam, k = 0.04) with half-life (t½) of 16.7 min (Midazolam, t½ = 17 min) and intrinsic clearance (CLint) of 3.30 mL/min/g (Midazolam, 3.23 mL/min/g). Gamitrinib showed negligible penetration across a monolayer of Caco-2 intestinal cells with an apparent permeability coefficient (P_app’_) of 1.90 nm/s in the A-to-B direction and 10.94 nm/s in the B-to-A direction with a P_app_ Efflux Ratio (ER) of 5.77 (Supplementary Table S1).

### Toxicity in Sprague-Dawley rats

**Figure 3.**
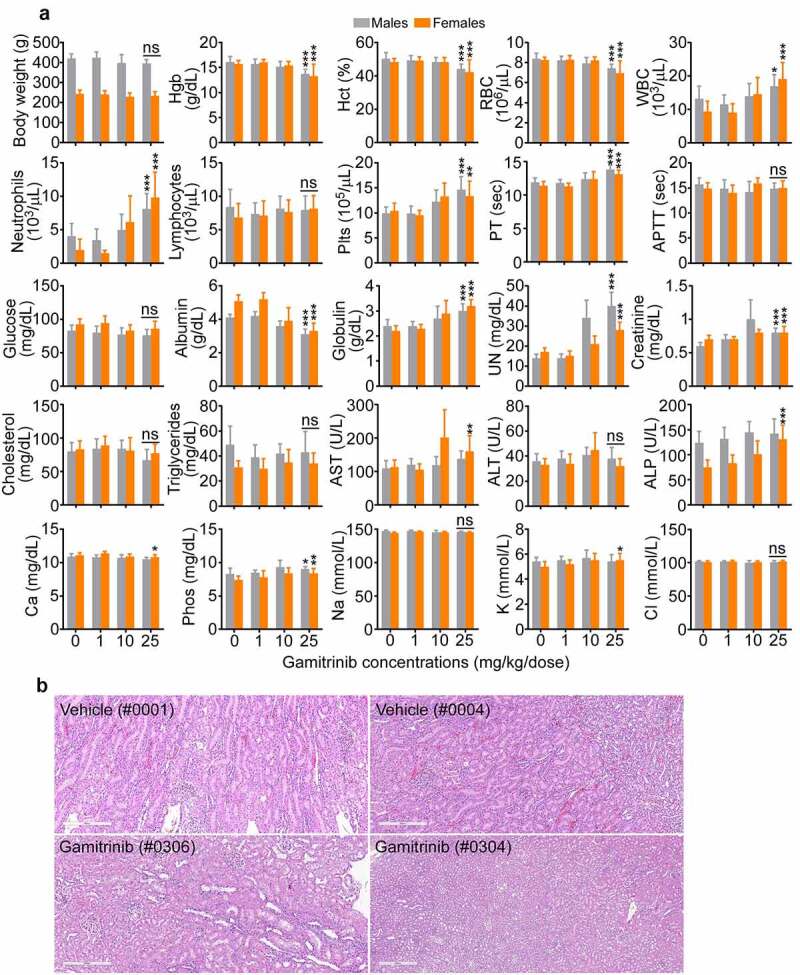
Gamitrinib toxicity in rats. (a) Males and females Sprague-Dawley rats were administered IV Gamitrinib at the indicated dose levels of 1, 10 and 25 mg/kg twice weekly for 29 d (dosing phase) weighed and blood samples collected at the end of the dosing phase were analyzed for the indicated clinical-chemistry parameters (mean ± SD). *, p = .01–0.03; **, p = .001–0.002; ***, p = .0004-<0.0001; ns, not significant. (b) Kidney histology of study rats (animal numbers in parentheses) administered vehicle or Gamitrinib IV (25 mg/kg/dose). Vehicle (#0001), normal kidney; Vehicle (#0004), minimal kidney degeneration/regeneration; Gamitrinib (#0306), slight kidney tubule degeneration/regeneration; Gamitrinib (#304), marked kidney tubule degeneration/regeneration. Representative images. Scale bar, 300 µm.

Male rats administered Gamitrinib IV (1 h infusion) at dose levels of 1, 10, or 25 mg/kg/dose twice weekly on d 1, 4, 8, 11, 15, 18, 22, 25, and 29 (dosing phase) exhibited a small, fully recoverable and not adverse reduction in mean body weights at 10 (−5.5%) or 25 (−5.7%) mg/kg/dose (Figure 3). Gamitrinib-related clinical observations involved animals administered ≥10 mg/kg/dose and included inguinal swelling, piloerection, hypoactivity, and sensitivity to touch at the infusion site. This is correlated with microscopic findings of mixed cell inflammation at the catheter/infusion site, which increased in incidence and/or severity in animals administered ≥10 mg/kg/dose (both sexes) and persisted through recovery. Alterations in clinical chemistry parameters, such as mildly to moderately higher neutrophil, and platelet (Plts) counts, minimally prolonged partial thromboplastin time (PT), lower albumin, higher globulin, and alkaline phosphatase concentrations were observed at the highest Gamitrinib dose level tested (Figure 3(a)) and likely related to inflammation, accompanied by histologic evidence of spleen and liver extramedullary hematopoiesis. Minimally to mildly higher serum urea nitrogen (UN) and creatinine concentrations in animals receiving Gamitrinib at 25 mg/kg/dose (Figure 3(a)) correlated with increased kidney weight and microscopic findings of tubular degeneration/regeneration (Figure 3(b)), which persisted to the end of the recovery phase. No effects on urinalysis or ophthalmic changes were identified. Gamitrinib-related mortality due to severe inflammation and marked hemorrhage at the infusion site occurred in two males, one female administered 25 mg/kg/dose and one male administered 10 mg/kg/dose. All other toxicity animals survived to their scheduled sacrifice. Gamitrinib-related mortality also occurred in two toxicokinetic females administered 10 mg/kg/dose and one toxicokinetic female administered 25 mg/kg/dose. Based on these findings, the non-observed adverse effect level (NOAEL) of Gamitrinib in rats is 1 mg/kg/dose, corresponding to C_max_ and AUC_0–24_ values of 87.1 ng/mL and 174 ng∙h/mL, respectively, on d 29 of dosing. Due to non-severely toxic effects or mortality in fewer than 10% of the animals administered 10 mg/kg/dose, the severely toxic dose in 10% of the animals (STD 10) is 10 mg/kg/dose, corresponding to C_max_ and AUC_0–24_ values of 311 ng/mL and 1300 ng∙h/mL, respectively, on d 29.

### Toxicity in beagle dogs

**Figure 4.**
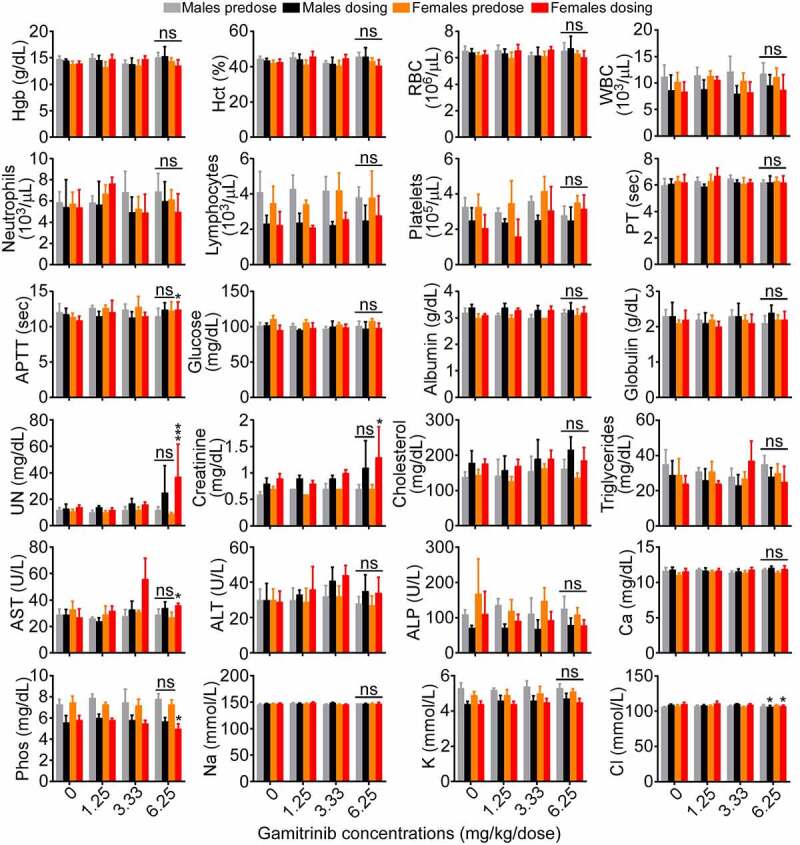
Gamitrinib toxicity in dogs. Male and female beagle dogs were administered IV Gamitrinib at the indicated dose levels of 1.25, 3.33 and 6.25 mg/kg twice weekly for 36 d and blood samples collected prior to the initiation of dosing (predose) and on d 36 of the dosing phase (dosing) were analyzed for the indicated clinical-chemistry parameters (mean ± SD). *, p = .01–0.04; ***, p = .001; ns, not significant.

Male and female beagle dogs administered IV Gamitrinib at dose levels of 1.25, 3.33, and 6.25 mg/kg/dose on d 1, 8, 15, 22, 29, and 36 of the dosing phase showed no alterations in body weight or other clinical observations (Supplementary Table S3). Bone marrow and liver parameters were unremarkable in all group levels, and only a trend of increased serum urea nitrogen and creatinine was observed in animals (both sexes) receiving the highest dose level of Gamitrinib of 6.25 mg/kg/dose (Figure 4). This correlated with microscopic findings of slight to moderate kidney tubular degeneration/regeneration, which was reversible during the recovery period. In addition, similar findings were present in one recovery sacrifice control male, making their relationship to Gamitrinib uncertain. No Gamitrinib-related changes in organ weight were observed and electrolyte, calcium, and phosphorus levels were unchanged in the various groups (Figure 4). Catheter and infusion site findings were similar in control and Gamitrinib-treated animals. Based on these findings, the NOAEL of Gamitrinib in dogs was 3.33 mg/kg/dose (C_max_, 560 ± 404 ng/mL; AUC_0–24_, 1740 ± 713 ng∙h/mL on d 36 of dosing, both sexes). In the absence of effects on the overt well-being of the animals and evidence of reversibility of Gamitrinib-related findings, 6.25 mg/kg/dose is considered the highest non-severely toxic dose (HNSTD). This dose level corresponds to C_max_ and AUC_0–24_ values of 1260 ± 556 ng/mL and 3290 ± 1090 ng∙h/mL, respectively (both sexes), on d 36 of the dosing phase.

## Discussion

In this study, we have shown that Gamitrinib, a first-in-class *mitocan* inhibitor of mitochondrial protein folding, can be synthesized as a clinical-grade material, formulated as a sterile, stable injectable suspension and administered IV to two animal species for up to 36 d. In line with its unique mechanism of action of subcellular organelle targeting, Gamitrinib drug-like properties differ from those of non-mitochondrial-targeted 17-AAG (Tanespimycin) with slower clearance, longer half-life, and unique metabolism without generation of 17-AG. Finally, prolonged IV administration of Gamitrinib is essentially unremarkable in beagle dogs and causes occasional inflammation at the infusion site and modest alterations of kidney function in Sprague-Dawley rats.

Although rewiring of metabolic pathways is a key tumor driver,^1^ and mitochondria provide an actionable, multifunctional therapeutic target,^7,8^ the (pre)clinical development of *mitocans* is mostly in infancy.^9^ The most visible conclusion of these studies is the encouraging preclinical safety of Gamitrinib, the first *mitocan* to disrupt a Hsp90 mitochondrial proteome in cancer.^19^ When administered to dogs, concentrations of Gamitrinib up to 12.8-fold higher than therapeutically active doses in mice (10 mg/kg) did not elicit the extensive alterations of bone marrow, liver, and gastrointestinal tract functions seen with non-mitochondrial targeted 17-AAG^37^ and its derivatives, 17-DMAG,^38^ and IPI-504.^39^ The only reportable observation in our study was that the highest dose level of Gamitrinib of 6.25 mg/kg caused only modest elevation of urea nitrogen and creatinine concentrations, with microscopic evidence of kidney tubule degeneration/regeneration, which was reversed during the 14-d recovery phase. The potential cardiac liability of Gamitrinib, prompted by the role of Hsp90 in hERG protein folding^40^ was also unremarkable. Gamitrinib IC_50_ for hERG conductance in patch-clamp studies (3.5 µM) was up to 20-fold times higher than the IC_50_ of inhibition of tumor growth observed in an NCI 60-cell line screen,^28^ and only 1 out of 10 animals exhibited a small, 7% prolongation of QTc interval (17 msec) fully reversed during the recovery phase. In the rat study, Gamitrinib concentrations (25 mg/kg) up to 5-fold higher than the efficacious antitumor dose in mice, produced only occasional inflammation at the infusion/catheter site, accompanied by mild elevation of urea nitrogen and evidence of kidney tubule degeneration/regeneration.

The molecular basis for the significantly more favorable safety of Gamitrinib compared to non-mitochondrial-targeted Hsp90 inhibitors^37–39^ remains to be elucidated. Previous data have suggested that Hsp90 may have greater affinity in tumors compared to normal tissues,^14^ which when combined with the selective accumulation of TRAP1 and Hsp90 in tumor mitochondria,^17^ may make the therapeutic inhibition of mitochondrial chaperones^15^ more efficient in cancer than normal tissues. It is also possible that the rapid intracellular transfer of Gamitrinib followed by its stable sequestration in mitochondria^30^ prevents a meaningful inhibition of cytosolic Hsp90, likely responsible for the toxicity of non-mitochondrial-targeted Hsp90 antagonists.^37–39^ This is consistent with other data showing that Gamitrinib treatment does not induce downregulation of Hsp90 client proteins,^31^ or a heat shock response, i.e. elevation of Hsp70, two hallmarks of Hsp90 inhibition in the cytosol.^27^

In addition to improved safety, Gamitrinib exhibited unique drug-like properties compared to 17-AAG. These included slower clearance (85.65 ± 5.85 mL/min/kg), much longer terminal-phase half-life (t_1/2_, 12.25 ± 1.55 h), and no generation of 17-AG, a key metabolite of 17-AAG processing.^36^ The structural basis for the increased Gamitrinib stability in vivo is currently not known. However, these data are reminiscent of the greater stability of 17-DMAG compared to 17-AAG,^41^ and it is possible that the presence of the triphenylphosphonium side chain reduces the oxidative metabolism of Gamitrinib,^41^ causing slower drug clearance and longer t_1/2_.

Despite the promise of Hsp90 as a hub of many oncogenic pathways,^42^ the clinical response to GA or non-GA Hsp90 antagonists has been disappointing.^27^ Although feasible, Hsp90-directed therapy has shown marginal, if any, patient responses as a single agent or in combination, hampered by unacceptable toxicity, including treatment-related deaths.^37–39^ These negative results have brought into question the validity of Hsp90 as a therapeutic target. Our data, combined with the preclinical anticancer activity of Gamitrinib,^31–35^ suggest a different scenario, where only a few, selected chaperone functions out of likely hundreds, are exploited for tumor growth.^42^ In this context, “untargeted,” global Hsp90 inhibition as previously pursued in the clinic,^27^ may exacerbate organ and tissue toxicity while paradoxically narrowing the therapeutic window. The pool of Hsp90 compartmentalized in tumor mitochondria,^19^ which escapes inhibition by “untargeted” Hsp90 antagonists,^28,30^ may provide one such key chaperone function exploited for tumor growth: preserving a multifunctional mitochondrial proteome highly vulnerable to oxidative and proteotoxic stress.

Based on the findings presented here, the feasibility and safety of Gamitrinib are currently being evaluated in a first-in-human, phase I clinical trial in patients with advanced cancer as part of a publicly funded academic effort (NCT04827810). In addition to validating mitochondrial proteostasis and Hsp90 as actionable therapeutic targets, the clinical development of Gamitrinib suggests that other mitochondrial functions exploited in cancer, such as one-carbon metabolism^43^ and DNA transcription,^44^ may also be druggable using a similar, “targeted” approach of subcellular drug delivery.^7,8^

## Supplementary Material

Supplemental Material

## References

[1] Vyas S, Zaganjor E, Haigis MC. 2016. Mitochondria and Cancer. Cell. 166:555–566. doi:10.1016/j.cell.2016.07.002. 27471965 PMC5036969

[2] Hanahan D, Weinberg RA. 2011. Hallmarks of cancer: the next generation. Cell. 144:646–674. doi:10.1016/j.cell.2011.02.013. 21376230

[3] Anderson RG, Ghiraldeli LP, Pardee TS. 2018. Mitochondria in cancer metabolism, an organelle whose time has come? Biochim Biophys Acta Rev Cancer. 1870:96–102. doi:10.1016/j.bbcan.2018.05.005. 29807044 PMC6420819

[4] Sabharwal SS, Schumacker PT. 2014. Mitochondrial ROS in cancer: initiators, amplifiers or an Achilles’ heel? Nat Rev Cancer. 14:709–721. doi:10.1038/nrc3803. 25342630 PMC4657553

[5] Galluzzi L, Bravo-San Pedro JM, Kroemer G. 2014. Organelle-specific initiation of cell death. Nat Cell Biol. 16:728–736. doi:10.1038/ncb3005. 25082195

[6] Caino MC, Altieri DC. 2016. Molecular pathways: mitochondrial reprogramming in tumor progression and therapy. Clin Cancer Res. 22:540–545. doi:10.1158/1078-0432.CCR-15-0460. 26660517 PMC4738153

[7] Fulda S, Galluzzi L, Kroemer G. 2010. Targeting mitochondria for cancer therapy. Nat Rev Drug Discov. 9:447–464. doi:10.1038/nrd3137. 20467424

[8] Vasan K, Werner M, Chandel NS. 2020. Mitochondrial metabolism as a target for cancer therapy. Cell Metab. 32:341–352. doi:10.1016/j.cmet.2020.06.019. 32668195 PMC7483781

[9] Macasoi I, Mioc A, Mioc M, Racoviceanu R, Soica I, Cheveresan A, Dehelean C, Dumitrascu V . Targeting mitochondria through the use of mitocans as emerging anticancer agents. Curr Med Chem. 2020;27:5730–5757. doi:10.2174/0929867326666190712150638. 31309878

[10] Prabhu VV, Morrow S, Rahman Kawakibi A, Zhou L, Ralff M, Ray J, Jhaveri A, Ferrarini I, Lee Y, Parker C, et al. 2020. ONC201 and imipridones: anti-cancer compounds with clinical efficacy. Neoplasia. 22:725–744. doi:10.1016/j.neo.2020.09.005. 33142238 PMC7588802

[11] Quintela-Fandino M, Morales S, Cortes-Salgado A, Manso L, Apala JV, Munoz M, Gasol Cudos A, Salla Fortuny J, Gion M, Lopez-Alonso A, et al. 2020. Randomized Phase 0/I trial of the mitochondrial inhibitor ME-344 or Placebo added to bevacizumab in early HER2-negative breast cancer. Clin Cancer Res. 26:35–45. doi:10.1158/1078-0432.CCR-19-2023. 31597662

[12] Reed GA, Schiller GJ, Kambhampati S, Tallman MS, Douer D, Minden MD, Yee KW, Gupta V, Brandwein J, Jitkova Y, et al. 2016. A Phase 1 study of intravenous infusions of tigecycline in patients with acute myeloid leukemia. Cancer Med. 5:3031–3040. doi:10.1002/cam4.845. 27734609 PMC5119957

[13] Alistar A, Morris BB, Desnoyer R, Klepin HD, Hosseinzadeh K, Clark C, Cameron A, Leyendecker J, D’Agostino R, Topaloglu U, et al. 2017. Safety and tolerability of the first-in-class agent CPI-613 in combination with modified FOLFIRINOX in patients with metastatic pancreatic cancer: a single-centre, open-label, dose-escalation, phase 1 trial. Lancet Oncol. 18:770–778. doi:10.1016/S1470-2045(17)30314-5. 28495639 PMC5635818

[14] Kamal A, Thao L, Sensintaffar J, Zhang L, Boehm MF, Fritz LC, Burrows FJ. 2003. A high-affinity conformation of Hsp90 confers tumour selectivity on Hsp90 inhibitors. Nature. 425:407–410. doi:10.1038/nature01913. 14508491

[15] Lin YF, Haynes CM. 2016. Metabolism and the UPR(mt). Mol Cell. 61:677–682. doi:10.1016/j.molcel.2016.02.004. 26942672 PMC4779188

[16] Ruggero D. 2013. Translational control in cancer etiology. Cold Spring Harb Perspect Biol. 5:a012336–a012336. doi:10.1101/cshperspect.a012336. 22767671 PMC3552512

[17] Kang BH, Plescia J, Dohi T, Rosa J, Doxsey SJ, Altieri DC. 2007. Regulation of tumor cell mitochondrial homeostasis by an organelle-specific Hsp90 chaperone network. Cell. 131:257–270. doi:10.1016/j.cell.2007.08.028. 17956728

[18] Cole A, Wang Z, Coyaud E, Voisin V, Gronda M, Jitkova Y, Mattson R, Hurren R, Babovic S, Maclean N, et al. 2015. Inhibition of the mitochondrial protease ClpP as a therapeutic strategy for human acute myeloid leukemia. Cancer Cell. 27:864–876. doi:10.1016/j.ccell.2015.05.004. 26058080 PMC4461837

[19] Chae YC, Angelin A, Lisanti S, Kossenkov AV, Speicher KD, Wang H, Powers JF, Tischler AS, Pacak K, Fliedner S, et al. 2013. Landscape of the mitochondrial Hsp90 metabolome in tumours. Nat Commun. 4:2139. doi:10.1038/ncomms3139. 23842546 PMC3732457

[20] Munch C, Harper JW. 2016. Mitochondrial unfolded protein response controls matrix pre-RNA processing and translation. Nature. 534:710–713. doi:10.1038/nature18302. 27350246 PMC4939261

[21] Lisanti S, Tavecchio M, Chae YC, Liu Q, Brice AK, Thakur ML, Languino L, Altieri D. 2014. Deletion of the mitochondrial chaperone TRAP-1 uncovers global reprogramming of metabolic networks. Cell Rep. 8:671–677. doi:10.1016/j.celrep.2014.06.061. 25088416 PMC4127146

[22] Basit F, van Oppen LM, Schockel L, Bossenbroek HM, van Emst-de Vries SE, Hermeling JC, Grefte S, Kopitz C, Heroult M, HGM Willems P, et al. 2017. Mitochondrial complex I inhibition triggers a mitophagy-dependent ROS increase leading to necroptosis and ferroptosis in melanoma cells. Cell Death Dis. 8:e2716. doi:10.1038/cddis.2017.133. 28358377 PMC5386536

[23] Siegelin MD, Dohi T, Raskett CM, Orlowski GM, Powers CM, Gilbert CA, Ross AH, Plescia J, Altieri DC. 2011. Exploiting the mitochondrial unfolded protein response for cancer therapy in mice and human cells. J Clin Invest. 121:1349–1360. doi:10.1172/JCI44855. 21364280 PMC3069780

[24] Chae YC, Caino MC, Lisanti S, Ghosh JC, Dohi T, Danial NN, Villanueva J, Ferrero S, Vaira V, Santambrogio L, et al. 2012. Control of tumor bioenergetics and survival stress signaling by mitochondrial HSP90s. Cancer Cell. 22:331–344. doi:10.1016/j.ccr.2012.07.015. 22975376 PMC3615709

[25] Altieri DC. 2013. Hsp90 regulation of mitochondrial protein folding: from organelle integrity to cellular homeostasis. Cell Mol Life Sci. 70:2463–2472. doi:10.1007/s00018-012-1177-0. 23052217 PMC3727647

[26] Feng Y, Nouri K, Schimmer AD. 2021. Mitochondrial ATP-dependent proteases-biological function and potential anti-cancer targets. Cancers. 13:2020. doi:10.3390/cancers13092020. 33922062 PMC8122244

[27] Neckers L, Workman P. 2012. Hsp90 molecular chaperone inhibitors: are we there yet? Clin Cancer Res. 18:64–76. doi:10.1158/1078-0432.CCR-11-1000. 22215907 PMC3252205

[28] Kang BH, Plescia J, Song HY, Meli M, Colombo G, Beebe K, Scroggins B, Neckers L, Altieri DC. 2009. Combinatorial drug design targeting multiple cancer signaling networks controlled by mitochondrial Hsp90. J Clin Invest. 119:454–464. doi:10.1172/JCI37613. 19229106 PMC2648691

[29] Gao Y, Tong H, Li J, Li J, Huang D, Shi J, Xia B. 2021. Mitochondria-targeted nanomedicine for enhanced efficacy of cancer therapy. Front Bioeng Biotechnol. 9:720508. doi:10.3389/fbioe.2021.720508. 34490227 PMC8418302

[30] Bryant KG, Chae YC, Martinez RL, Gordon JC, Elokely KM, Kossenkov AV, Grant S, Childers WE, Abou-Gharbia M, Altieri DC, et al. 2017. A mitochondrial-targeted purine-based HSP90 antagonist for leukemia therapy. Oncotarget. 8:112184–112198. doi:10.18632/oncotarget.23097. 29348817 PMC5762502

[31] Kang BH, Siegelin MD, Plescia J, Raskett CM, Garlick DS, Dohi T, Lian JB, Stein GS, Languino LR, Altieri DC, et al. 2010. Preclinical characterization of mitochondria-targeted small molecule hsp90 inhibitors, gamitrinibs, in advanced prostate cancer. Clin Cancer Res. 16:4779–4788. doi:10.1158/1078-0432.CCR-10-1818. 20876793 PMC2948625

[32] Kang BH, Tavecchio M, Goel HL, Hsieh CC, Garlick DS, Raskett CM, Lian JB, Stein GS, Languino LR, Altieri DC, et al. 2011. Targeted inhibition of mitochondrial Hsp90 suppresses localised and metastatic prostate cancer growth in a genetic mouse model of disease. Br J Cancer. 104:629–634. doi:10.1038/bjc.2011.9. 21285984 PMC3049604

[33] Zhang G, Frederick DT, Wu L, Wei Z, Krepler C, Srinivasan S, Chae YC, Xu X, Choi H, Dimwamwa E, et al. 2016. Targeting mitochondrial biogenesis to overcome drug resistance to MAPK inhibitors. J Clin Invest. 126:1834–1856. doi:10.1172/JCI82661. 27043285 PMC4855947

[34] Ghosh JC, Siegelin MD, Vaira V, Faversani A, Tavecchio M, Chae YC, Lisanti S, Rampini P, Giroda M, Caino MC, et al. 2015. Adaptive mitochondrial reprogramming and resistance to PI3K therapy. J Natl Cancer Inst. 107. doi:10.1093/jnci/dju502. PMC4565533 25650317

[35] Karpel-Massler G, Ishida CT, Bianchetti E, Shu C, Perez-Lorenzo R, Horst B, Banu M, Roth KA, Bruce JN, Canoll P, et al. 2017. Inhibition of mitochondrial matrix chaperones and antiapoptotic Bcl-2 family proteins empower antitumor therapeutic responses. Cancer Res. 77:3513–3526. doi:10.1158/0008-5472.CAN-16-3424. 28522750 PMC5503474

[36] Egorin MJ, Zuhowski EG, Rosen DM, Sentz DL, Covey JM, Eiseman JL. 2001. Plasma pharmacokinetics and tissue distribution of 17-(allylamino)-17-demethoxygeldanamycin (NSC 330507) in CD2F1 mice1. Cancer Chemother Pharmacol. 47:291–302. doi:10.1007/s002800000242. 11345645

[37] Solit DB, Ivy SP, Kopil C, Sikorski R, Morris MJ, Slovin SF, Kelly WK, DeLaCruz A, Curley T, Heller G, et al. 2007. Phase I trial of 17-allylamino-17-demethoxygeldanamycin in patients with advanced cancer. Clin Cancer Res. 13:1775–1782. doi:10.1158/1078-0432.CCR-06-1863. 17363532 PMC3203693

[38] Pacey S, Wilson RH, Walton M, Eatock MM, Hardcastle A, Zetterlund A, Arkenau H-T, Moreno-Farre J, Banerji U, Roels B, et al. 2011. A phase I study of the heat shock protein 90 inhibitor alvespimycin (17-DMAG) given intravenously to patients with advanced solid tumors. Clin Cancer Res. 17:1561–1570. doi:10.1158/1078-0432.CCR-10-1927. 21278242 PMC3060938

[39] Oh WK, Galsky MD, Stadler WM, Srinivas S, Chu F, Bubley G, Goddard J, Dunbar J, Ross RW. 2011. Multicenter phase II trial of the heat shock protein 90 inhibitor, retaspimycin hydrochloride (IPI-504), in patients with castration-resistant prostate cancer. Urology. 78:626–630. doi:10.1016/j.urology.2011.04.041. 21762967 PMC3166448

[40] Ficker E, Dennis AT, Wang L, Brown AM. 2003. Role of the cytosolic chaperones Hsp70 and Hsp90 in maturation of the cardiac potassium channel HERG. Circ Res. 92:e87–100. doi:10.1161/01.RES.0000079028.31393.15. 12775586

[41] Zheng N, Zou P, Wang S, Sun D. 2011. In vitro metabolism of 17-(dimethylaminoethylamino)-17-demethoxygeldanamycin in human liver microsomes. Drug Metab Dispos. 39:627–635. doi:10.1124/dmd.110.036418. 21177985 PMC3063720

[42] Taipale M, Jarosz DF, Lindquist S. 2010. HSP90 at the hub of protein homeostasis: emerging mechanistic insights. Nat Rev Mol Cell Biol. 11:515–528. doi:10.1038/nrm2918. 20531426

[43] Zhao LN, Bjorklund M, Caldez MJ, Zheng J, Kaldis P. 2021. Therapeutic targeting of the mitochondrial one-carbon pathway: perspectives, pitfalls, and potential. Oncogene. 40:2339–2354. doi:10.1038/s41388-021-01695-8. 33664451

[44] Bonekamp NA, Peter B, Hillen HS, Felser A, Bergbrede T, Choidas A, Horn M, Unger A, Di Lucrezia R, Atanassov I, et al. 2020. Small-molecule inhibitors of human mitochondrial DNA transcription. Nature. 588:712–716. doi:10.1038/s41586-020-03048-z. 33328633

