# Supplementary material for: Feasibility and safety of targeting mitochondria for cancer therapy – preclinical characterization of gamitrinib, a first-in-class, mitochondriaL-targeted small molecule Hsp90 inhibitor
Source: Cancer Biol Ther. 2022 Feb 6;23(1):117–26. doi: 10.1080/15384047.2022.2029132 (PMC8820820; doi:10.1080/15384047.2022.2029132)
Supplement: Supplemental Material [file KCBT_A_2029132_SM5395.zip › supplementary/SUPPLEMENTARY INFORMATION Rev Ms KBT_2021_0626.docx]

**FEASIBILITY AND SAFETY OF TARGETING MITOCHONDRIA FOR CANCER THERAPY –PRECLINICAL CHARACTERIZATION OF GAMITRINIB, A FIRST-IN-CLASS, MITOCHONDRIAL-TARGETED SMALL MOLECULE Hsp90 INHIBITOR**

Umar Hayat, Gary T. Elliott, Anthony J. Olszanski and Dario C. Altieri

**SUPPLEMENTARY INFORMATION**

**SUPPLEMENTARY MATERIALS AND METHODS**

**Chemicals and reagents**. Quercetin, ketoconazole, phenacetin, bupropion, paclitaxel, diclofenac, midazolam, testosterone, tolbutamide, β-nicotinamide adenine dinucleotide phosphate sodium salt hydrate (NADP), D-glucose 6-phosphate sodium salt (G6P), glucose-6-phosphate dehydrogenase (G6PDH), digoxin, sucrose, dextrose, propranolol, lucifer yellow (LY), warfarin, HEPES, D-glucose, terfenadine, polysorbate 80 and dimethyl sulfoxide (DMSO) were obtained from Sigma-Aldrich (St. Louis, MO). Fluvoxamine, tranylcypromine, clopidogrel, and sulfaphenazole were obtained from Cayman Chemicals (Ann Arbor, MI). Lecithin was purchased from Lipoid (Lipoid S100). 17-amino-geldanamycin (17-AG) was obtained from Toronto Research Chemicals (Toronto, Canada). Potassium phosphate buffer (KPi) was prepared at 100 mM, pH 7.4. NADPH regenerating system, cofactor (5X) was prepared with NADP (1.7 mg/mL, 2.22 mM), G6P (7.8 mg/mL, 27.6 mM) and G6DPH (2.0 U/mL) in 100 mM KPi. Human plasma and pooled human liver microsomes were obtained from Bioreclamation IVT (Baltimore, MD). HBSS, acetonitrile, BupH phosphate buffered saline packs and rapid equilibrium dialysis (RED) devices were obtained from Thermo Fisher Scientific (Waltham, MA). Stop solution was prepared with ACN containing 100 ng/mL tolbutamide.

**Plasma protein binding**. Gamitrinib or control stock solutions (5 mM) were prepared in DMSO, and further diluted in DMSO/water (1:1, *v*/*v*) to 0.2 mg/mL. Spiked plasma samples were prepared by adding 12 µL of each working solution into 1.2 mL of blank plasma to a final concentration of 2 μg/mL. Plasma protein binding incubations were performed in triplicate by adding 500 µL of PBS into each white chamber and 300 µL of spiked plasma into each red chamber of the RED device. The device was covered with a sealing tape and placed on an orbital shaker at a shaking speed of 100 rpm at 37°C in 5% CO_2_. After a 4-h incubation, a 40 µL plasma sample was transferred from each red chamber to the wells of a 96-well plate containing 160 µL of PBS. A 160 µL PBS aliquot from each white chamber was transferred to the wells of the same plate containing 40 µL of blank plasma. After matching the matrix for all samples, 400 µL aliquots of stop solution were added to each well of the 96-well sample plate. The plate was vortexed for 3 min at 1700 rpm followed by centrifugation at 3500 rpm for 15 min. A 100‑µL aliquot of supernatant from each well was transferred to a new 96-well sample plate and further mixed with 100 µL of Milli-Q (ultrapure) water before analysis by liquid chromatography–tandem mass spectrometry (LC-MS/MS).

**CYP inhibition in liver microsomes**. Working solutions of Gamitrinib at concentrations of 0, 0.033, 0.1, 0.33, 1, 3.3, 10 and 33 μM were incubated with human liver microsomes (0.1 mg/mL) with an NADPH regenerating system. Positive controls were added to human liver microsomes in parallel reactions with serial concentrations of 0, 0.033, 0.1, 0.33, 1, 3.3, 10, 33 μM (clopidogrel, CYP2B6) or 0.0033, 0.01, 0.033, 0.1, 0.33, 1, 3.3 μM (fluvoxamine, CYP1A2; tranylcypromine, CYP2A6; quercetin, CYP2C8; sulfaphenazole, CYP2C9; ketoconazole, CYP3A4). After addition of prototypical CYP substrates in 250 µL, samples were incubated for 10 min (CYP1A2, CYP2B6, CYP2C9, and CYP3A4) or 20 min (CYP2C8) at 37°C in 5% CO_2_. After addition of 250 µL stop solution, the plate was vortexed for 3 min at 1700 rpm and centrifuged at 3500 rpm for 15 min. Samples were analyzed for metabolite formation by LC-MS/MS. The results were expressed using the peak area ratio of analyte to internal standard, based on the calibration curves. The inhibitor concentration that resulted in 50% inhibition (IC_50_) of enzyme activity was calculated by fitting the % control activity *vs*. concentration to the following equation:

*y*=$\frac{Range}{\left[ 1+\left( \frac{x}{IC50} \right)^{s} \right]}$

Where *Range*=fitted uninhibited value, *s*=slope factor, *x*=inhibitor concentration, *y*=% control activity.

**Ion channel screening**. To test the effect of Gamitrinib (10 μM) on channel conductance, human Nav1.5 currents were evoked by stepping from a holding potential of -120 mV to -20 mV for 150 msec (50 msec inter-pulse interval) for a total of 26 pulses. The parameters measured were the maximum inward current evoked on stepping to -20 mV from the 1^st^ and 26^th^ pulse. Human Kv4.3/KChIP2 currents were evoked from a holding potential of -80 mV by a series of four 500 msec pulses to 0 mV using a 1000 msec interval between pulses. The parameter measured was the amplitude of the outward current 50 msec after the onset of the 4^th^ voltage step to 0 mV. Human Cav1.2 currents were evoked by 2 pulses to -10 mV from a holding potential of -100 mV. The parameters measured were the maximum inward currents evoked on stepping to -10 mV from the holding potential of -100 mV for the 1^st^ and 2^nd^ pulse. Human Kv1.5 currents were evoked by a single pulse from a holding potential of -80 mV to a potential of 0 mV for 4 sec before returning to -80 mV. The parameters measured were the maximum amplitude of outward currents evoked at the beginning of the voltage pulse and at the end of the voltage step from -80 mV to 0 mV. Human KCNQ1/minK currents were evoked by a single pulse delivered from a holding potential of -80 mV to +60 mV for 4 sec before returning to -80 mV. The parameter measured was the maximum outward current evoked on stepping to +60 mV from the holding potential of -80 mV. hERG currents were evoked by a three-pulse protocol where voltage was first stepped to +40 mV for two sec from a holding potential of –80 mV to inactivate hERG channels. The voltage is then stepped back to –50 mV for two sec to evoke a tail current prior to returning to the holding potential for 1 sec. The parameter measured was the amplitude of the 3^rd^ pulse tail current upon stepping back to -50 mV after the step to +40 mV. Human HCN4 currents were evoked by a single pulse from a holding potential of -30 mV to a potential of -110 mV for 4 sec prior to returning to -30 mV. The parameter measured was the maximum inward current evoked upon stepping to -110 mV from the holding potential of -30 mV. Human Kir2.1 currents were evoked from a holding potential of -20 mV by a series of 10 500 msec pulses to -120 mV using a 200 msec interval between pulses. The parameters measured were the amplitudes of the instantaneous inward currents evoked on stepping to -120 mV for the 1^st^ pulse and the maximum inward current at the end of the 10^th^ hyperpolarizing pulse. For all experiments, data were filtered for seal quality, seal drop, and current amplitude.

**Patch clamp recording**. The extracellular solution for whole cell patch clamp recordings was 137 mM NaCl, 1.2 mM MgCl_2_, 5.4 mM KCl, 10 mM glucose, 10 mM HEPES and 2 mM CaCl_2_ (305 mOsm), pH 7.4. The intracellular solution was 140 mM KCl, 2.1 mM MgCl, 5 mM EGTA, 10 mM HEPES and 5 mM Na_2_ATP (295 mOsm), pH 7.2. For electrophysiology recording, a micropipette was pulled from borosilicate glass with the pipette tip resistance between 3 ~ 5 MΩ. For each experiment, polyclonal HEK293 cells stably transfected with hERG cDNA and maintained in 250 μg/ml Geneticin (G418) were plated on 35 mm dishes on the microscope stage and a commercial patch clamp amplifier was used for the whole cell recordings. The tail currents were evoked at 22°C once every 30 sec by a 3 sec-50 mV repolarizing pulse following a 2 sec +50 mV depolarizing pulse with a hold voltage of -80 mV. A 50 msec depolarized pulse to -50 mV at the beginning of the voltage protocol served as a baseline for calculating the amplitude of the peak tail current. The hERG currents were allowed to stabilize over a 3 min period in the presence of vehicle alone prior to Gamitrinib application. The cells were kept in the test solution until the peak tail current was stable (<5% change) for ~5 sweeps or for a maximum of 6 min, whichever came first. Peak tail amplitudes were plotted as a function of the sweep number. Five peak tail current measurements at the steady state before Gamitrinib application were averaged and used as the control current amplitude. Four or five peak tail current measurements at the steady state after Gamitrinib application were averaged and used as the remaining current amplitude after inhibition by the test article. The % inhibition of the test article was calculated from the following equation:

% inhibition = 1- (remaining current amplitude)/ (control current amplitude) ∗100.

**Electrocardiography studies**. Electrocardiograms (ECGs) were obtained from unanesthetized beagle dogs administered Gamitrinib at dose levels of 1.25 mg/kg/dose (3 males and 3 females), 3.33 mg/kg/dose (3 males and 3 females) and 6.25 mg/kg/dose (5 males and 5 females) once during the predose phase, 1 to 2 h post end of infusion on d 32 of the dosing phase, and on d 11 of the recovery phase. At each time point, eight-lead ECGs were continuously recorded for at least 30 sec ^1^. At least five consecutive ECG waveforms and the associated RR intervals deemed most representative were selected for quantitative analysis at each time point. The ECG interval measurements (PR, QRS, QT, QTc, RR, and heart rate) were made on a single lead. The measured waveforms at each time point were averaged and reported. The QTc interval was calculated using the Fridericia (QT/[RR]^1/3^) method ^2^. The RR interval was used to derive the heart rate and in QT interval correction. Qualitative assessments included normal sinus rhythm variations, abnormal sinus rhythms, conductance or repolarization abnormalities, bradycardia, and tachycardia. Group comparisons of ECG data (PR, QRS, QT, QTc, and heart rate) were analyzed using ANOVA.

**Bidirectional Caco2 cell monolayer permeability**. Caco2 cells (2x10^4^/well) were seeded onto apical chambers of a 24-multiwell insert plate and incubated at 37°C in 5% CO_2_. After 21 d, the transepithelial electrical resistance (TEER) in ohms was read for each insert well as a quality control for monolayer integrity (>900 ohms or 300 ohms cm^2^). The cell monolayers were incubated with 400 µL transport buffer on the apical (A) and 1.2 mL on the basolateral (B) sides for 30 min. After preincubation, dosing solution was added to the apical (400 µL, A-to-B, donor) or basolateral sides (1.2 mL, B-to-A, donor) or receiving buffer to the apical (400 µL, B-to-A, receiver) or basolateral sides (1.2 mL, A-to-B, receiver) for 90 min at 37°C in 5% CO_2_. At the end of the incubation, 300 µL samples from A and B sides were transferred to a receiving plate, centrifuged for 15 min at 3500 rpm and analyzed by LC-MS/MS. The integrity of each monolayer was assessed with Lucifer Yellow (LY) at Em 430 nm and Ex 540 nm (batch results, 0.41; acceptance criteria, <1% of dosing solution) and TEER values (batch results, 443; acceptance criteria, >300). P_app’_A>B (nm/s) was calculated with Equation 1.

Equation 1:

$$Papp=\frac{Vr\cdot Cr}{A\cdot t\cdot Cdo}$$

where Cr is concentration in receiver well, Cd0 is initial dosing concentration, Vr is the receiver well volume; A is the membrane surface area (0.3 cm^2^) and T is the incubation time (120 min*60s/min).

Papp Efflux Ratio (ER) was calculated with Equation 2.

Equation 2:

$$ER=\frac{Papp,B>A}{Papp,A>B}$$

Propranolol (high permeability marker, non-P-gp substate) and digoxin (P-gp substrate) were used as controls.

**SUPPLEMENTARY REFERENCES**

1. Detweiler DK. Electrocardiographic monitoring in toxicological studies: principles and interpretations. Adv Exp Med Biol 1983; 161:579-607.

2. Toyoshima S, Kanno A, Kitayama T, Sekiya K, Nakai K, Haruna M, et al. QT PRODACT: in vivo QT assay in the conscious dog for assessing the potential for QT interval prolongation by human pharmaceuticals. J Pharmacol Sci 2005; 99:459-71.


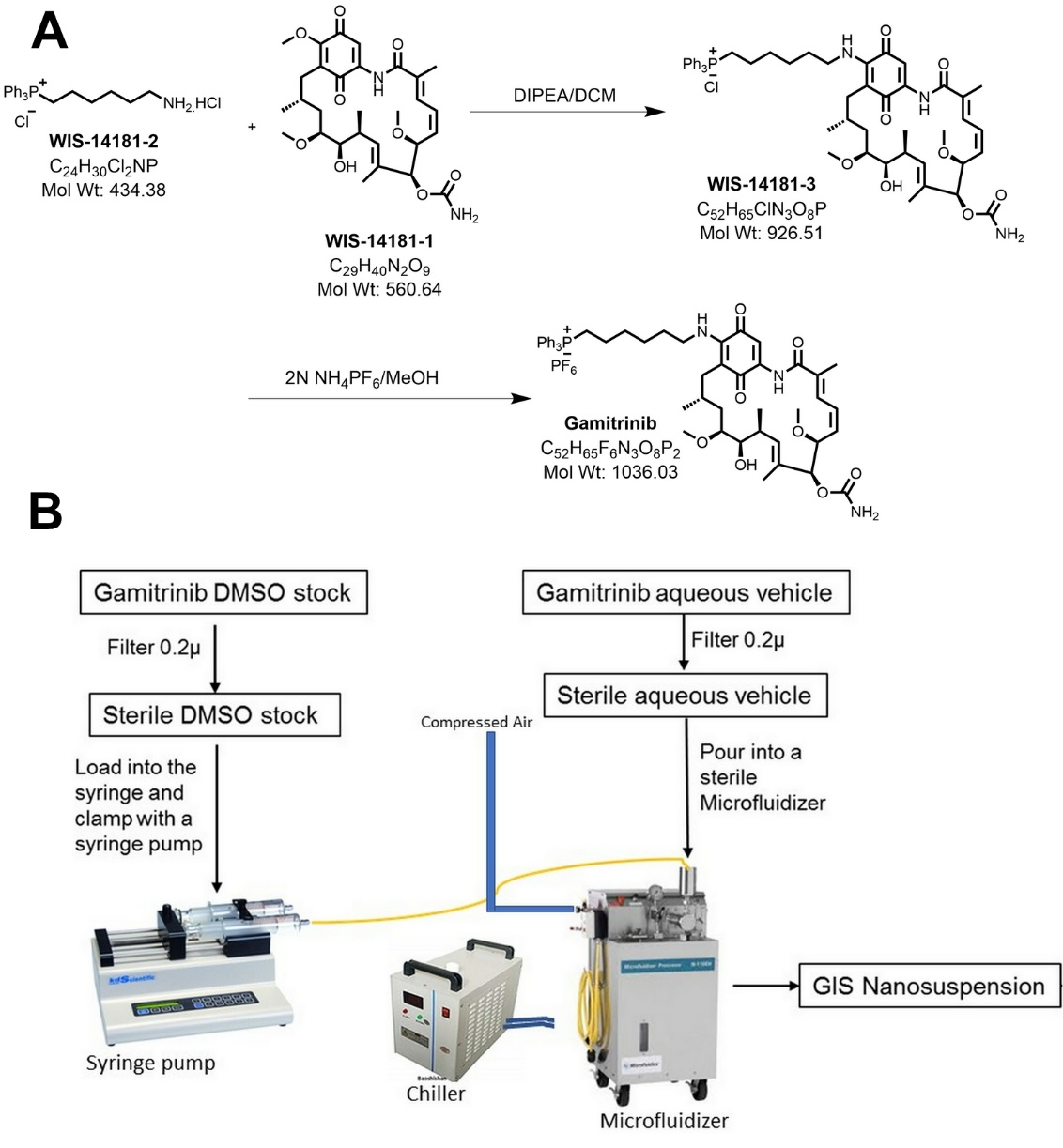
**SUPPLEMENTARY FIGURE LEGENDS**

**Supplementary Figure S1**. Gamitrinib. (a) Stepwise chemical synthesis of Gamitrinib linking the Hsp90 ATPase inhibitor 17-AAG to the mitochondrial import carrier, triphenylphosphonium via a hexylamine linker. (b) Workflow of Gamitrinib drug product with preparation of a GMP Gamitrinib Injectable Suspension (GIS, particle size, <200 nm) by microfluidization.


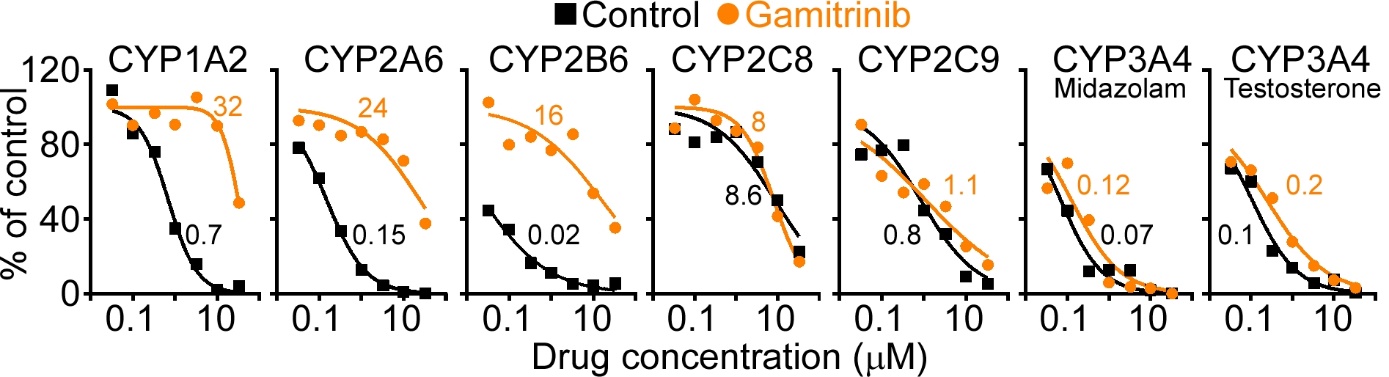


**Supplementary Figure S2**. CYP inhibition. Increasing concentrations of Gamitrinib or relevant control were incubated with the indicated CYP isoforms and analyzed for % inhibition. The individual IC_50_ values of Gamitrinib (yellow) or control (black) are indicated per each CYP tested.


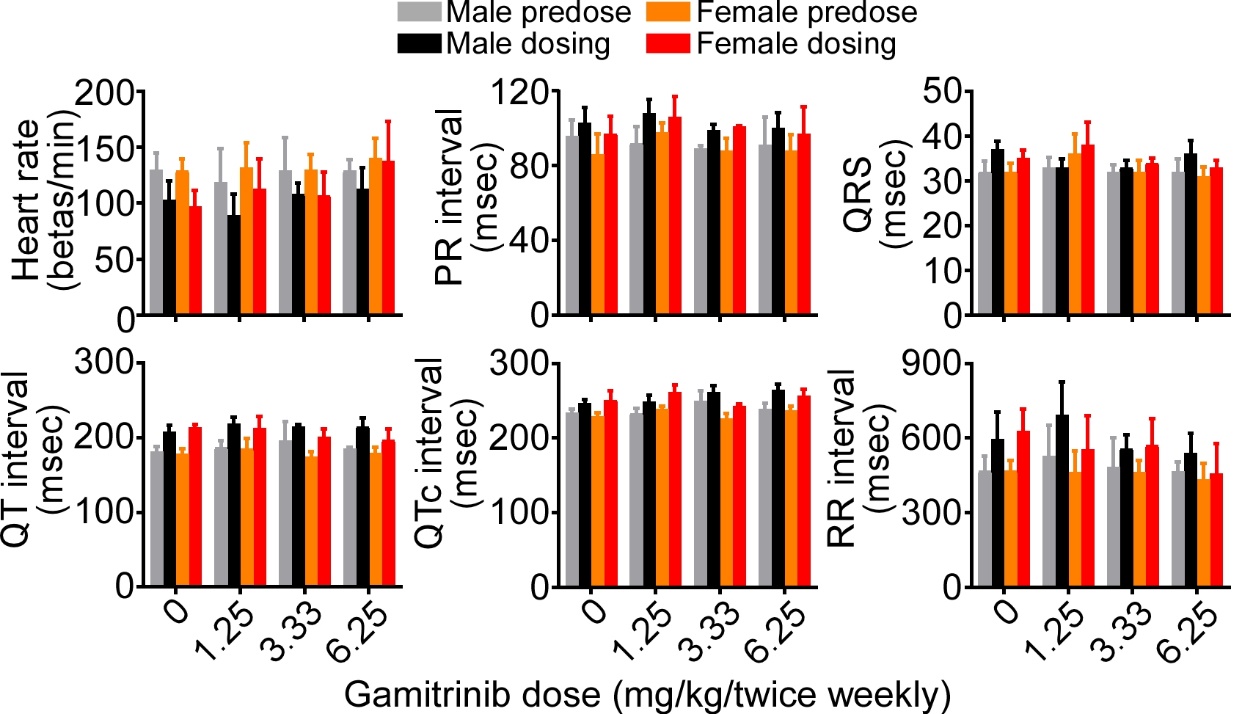


**Supplementary Figure S3**. Electrophysiologic studies. Male and female beagle dogs were administered IV Gamitrinib at the indicated dose levels of 1.25, 3.33 and 6.25 mg/kg twice weekly and electrocardiograms (ECGs) were obtained from unanesthetized animals once during the predose phase (predose) and 1 to 2 h post end of infusion on d 32 of the dosing phase (dosing). Mean±SD.

**SUPPLEMENTARY TABLES**

**Supplementary Table S1**. Gamitrinib permeability in a Caco2 intestinal cell monolayer assay

| Test Compound | P_app_, A>B (nm/s) | P_app_, B>A (nm/s) | P_app_ Efflux Ratio (ER) | P_exact_,A>B (nm/s) | P_exact_,B>A (nm/s) | P_exact_ Efflux Ratio |
| --- | --- | --- | --- | --- | --- | --- |
| Gamitrinib | 1.90 | 10.94 | 5.77 | 1.97 | 10.92 | 5.54 |
| Propranolol | 186.12 | 162.49 | 0.87 | 209.35 | 170.12 | 0.81 |
| Digoxin | 5.47 | 114.80 | 21.00 | 4.13 | 87.64 | 21.21 |

**Supplementary Table S2**. Time course of Gamitrinib and 17-AG plasma concentrations after IV administration of Gamitrinib (5 mg/kg) to Sprague-Dawley rats

| Time (min) | Animal No. | Collection Day | Dose (mg/kg) | Gamitrinib (ng/mL) | 17-AG (ng/mL) |  |
| --- | --- | --- | --- | --- | --- | --- |
|  |  |  |  |  |  |  |
| 5 | 1 | 1 | 5 | 622.199 | BLOQ |  |
| 15 | 1 | 1 | 5 | 180.951 | 0.561 |  |
| 30 | 1 | 1 | 5 | 106.381 | BLOQ |  |
| 60 | 1 | 1 | 5 | 71.445 | BLOQ |  |
| 120 | 1 | 1 | 5 | 46.657 | BLOQ |  |
| 240 | 1 | 1 | 5 | 36.816 | BLOQ |  |
| 1440 | 1 | 2 | 5 | 11.946 | BLOQ |  |
| 5 | 2 | 1 | 5 | 526.229 | 1.519 |  |
| 15 | 2 | 1 | 5 | 211.686 | BLOQ |  |
| 30 | 2 | 1 | 5 | 114.230 | BLOQ |  |
| 60 | 2 | 1 | 5 | 65.903 | BLOQ |  |
| 120 | 2 | 1 | 5 | 43.125 | BLOQ |  |
| 240 | 2 | 1 | 5 | 34.776 | BLOQ |  |
| 1440 | 2 | 2 | 5 | 10.511 | BLOQ |  |
| 5 | 3 | 1 | 5 | 674.432 | 1.113 |  |
| 15 | 3 | 1 | 5 | 128.663 | BLOQ |  |
| 30 | 3 | 1 | 5 | 67.204 | BLOQ |  |
| 60 | 3 | 1 | 5 | 55.991 | BLOQ |  |
| 120 | 3 | 1 | 5 | 29.914 | BLOQ |  |
| 240 | 3 | 1 | 5 | 28.905 | BLOQ |  |
| 1440 | 3 | 2 | 5 | 10.365 | BLOQ |  |

BLOQ, below limit of quantification.

**Supplementary Table S3**. Body weight alterations (Kg) in beagle dogs after Gamitrinib IV infusion (dosing phase). N=number of animals.

| Days | MALES Gamitrinib doses (IV infusion) | | | |
| --- | --- | --- | --- | --- |
|  | 0 mg/kg | 1.25 mg/kg | 3.33 mg/kg | 6.25 mg/kg |
| 1 | 10.1±0.83 (N=5) | 10.3±0.91 (N=3) | 10.1±1.1 (N=3) | 9.9±0.83 (N=5) |
| 8 | 10±0.75 (N=5) | 10.2±1.04 (N=3) | 10±1.04 (N=3) | 9.8±0.85 (N=5) |
| 15 | 9.9±0.66 (N=5) | 10.1±0.93 (N=3) | 9.9±1.15 (N=3) | 9.5±0.57 (N=5) |
| 22 | 10.1±0.73 (N=5) | 10.3±0.69 (N=3) | 10.2±1.21 (N=3) | 9.2±0.57 (N=5) |
| 29 | 10±0.66 (N=5) | 10.3±0.75 (N=3) | 10.1±1.21 (N=3) | 9.2±0.46 (N=5) |
| 36 | 10.1±0.76 (N=5) | 10.5±0.52 (N=3) | 10±1.36 (N=3) | 9.1±0.58 (N=5) |

| Days | FEMALES Gamitrinib doses (IV infusion) | | | |
| --- | --- | --- | --- | --- |
|  | 0 mg/kg | 1.25 mg/kg | 3.33 mg/kg | 6.25 mg/kg |
| 1 | 7.5±0.92 (N=5) | 7.9±1.34 (N=3) | 7.6±0.9 (N=3) | 7.7±0.57 (N=5) |
| 8 | 7.4±0.89 (N=5) | 7.8±1.49 (N=3) | 7.6±0.9 (N=3) | 7.7±0.62 (N=5) |
| 15 | 7.3±0.93 (N=5) | 7.8±1.64 (N=3) | 7.6±1.06 (N=3) | 7.4±0.54 (N=5) |
| 22 | 7.3±1.03 (N=5) | 8±1.53 (N=3) | 7.5±0.75 (N=3) | 7.2±0.6 (N=5) |
| 29 | 7.5±0.94 (N=5) | 8.2±1.42 (N=3) | 7.6±0.7 (N=3) | 7.3±0.58 (N=5) |
| 36 | 7.6±0.97 (N=5) | 8.4±1.55 (N=3) | 7.5±0.8 (N=3) | 7.3±0.59 (N=5) |
